# Diagnostic utility of next-generation sequencing-based panel testing in 543 patients with suspected skeletal dysplasia

**DOI:** 10.1186/s13023-021-02025-7

**Published:** 2021-10-09

**Authors:** Alicia Scocchia, Tiia Kangas-Kontio, Melita Irving, Matti Hero, Inka Saarinen, Liisa Pelttari, Kimberly Gall, Satu Valo, Johanna M. Huusko, Jonna Tallila, Johanna Sistonen, Juha Koskenvuo, Tero-Pekka Alastalo

**Affiliations:** 1 Blueprint Genetics Inc, Seattle, WA USA; 2 Blueprint Genetics Oy, Espoo, Finland; 3 Department of Clinical Genetics, Guy’s and St. Thomas’ NHS Trust, London, UK; 4 New Children’s Hospital, Pediatric Research Center, Helsinki University Hospital, Helsinki, Finland

**Keywords:** Skeletal dysplasia, Skeletal disorders, Next-generation sequencing, Multi-gene panel, Molecular diagnostics, Genetic diagnostics, Prenatal genetic testing, Copy number variant analysis

## Abstract

**Background:**

Skeletal dysplasia is typically diagnosed using a combination of radiographic imaging, clinical examinations, and molecular testing. Identifying a molecular diagnosis for an individual with a skeletal dysplasia can lead to improved clinical care, guide future medical management and treatment, and inform assessment of risk for familial recurrence. The molecular diagnostic utility of multi-gene panel testing using next-generation sequencing (NGS) has not yet been characterized for an unselected population of individuals with suspected skeletal dysplasia. In this study, we retrospectively reviewed patient reports to assess the diagnostic yield, reported variant characteristics, impact of copy number variation, and performance in prenatal diagnostics of panel tests for variants in genes associated with skeletal dysplasia and growth disorders.

**Results:**

Clinical reports of consecutive patients with a clinical indication of suspected skeletal dysplasia who underwent panel testing were examined. The 543 patients included in the study submitted samples for diagnostic genetic testing with an indication of suspected skeletal dysplasia or growth disorder and received one of three nested panel tests. A molecular diagnosis was established in 42.0% of patients (n = 228/543). Diagnostic variants were identified in 71 genes, nearly half of which (n = 35, 49.3%) contributed uniquely to a molecular diagnosis for a single patient in this cohort. Diagnostic yield was significantly higher among fetal samples (59.0%, n = 52/88) than postnatal samples (38.7%, n = 176/455; z = 3.55, *p* < 0.001). Diagnostic variants in fetal cases were identified across 18 genes. Thirteen diagnostic CNVs were reported, representing 5.7% of diagnostic findings and ranging in size from 241-bp to whole chromosome aneuploidy. Additionally, 11.4% (36/315) of non-diagnostic patient reports had suspicious variants of unknown significance (VUS), in which additional family studies that provide segregation data and/or functional characterization may result in reclassification to likely pathogenic.

**Conclusions:**

These findings demonstrate the utility of panel testing for individuals with a suspected skeletal dysplasia or growth disorder, with a particularly high diagnostic yield seen in prenatal cases. Pursuing comprehensive panel testing with high-resolution CNV analysis can provide a diagnostic benefit, given the considerable phenotype overlap amongst skeletal dysplasia conditions.

**Supplementary Information:**

The online version contains supplementary material available at 10.1186/s13023-021-02025-7.

## Background

Skeletal dysplasia conditions encompass over 450 clinically and genetically heterogeneous conditions involving abnormalities of cartilage and bone [1]. Suspicion of skeletal dysplasia often results from observing a difference in growth patterns, which may be recognized prenatally, or disproportionate short stature. Given significant phenotype overlap amongst these rare conditions, arriving at a precise diagnosis may require significant medical evaluation by several specialists with distinct expertise [2–4]. These conditions are typically diagnosed using a combination of radiographic imaging, clinical assessment, and molecular testing [5]. Clinical and radiographical phenotyping often involves assessment of medical and family histories, anthropomorphic measurements and growth trajectory, skeletal survey X-rays for dysplasia investigations via X-ray imaging, and may also involve histological evaluations from bone biopsy or surgical specimens [3]. Obstetric ultrasound [4] and fetal MRI [6] can be used for prenatal investigations. Molecular testing is used to identify the precise genetic variation responsible for an affected individual’s clinical features, referred to as the molecular diagnosis, within the context of clinical and radiographic evidence.

Identifying a molecular diagnosis for an individual with a skeletal dysplasia can lead to improved clinical care, including guiding medical management or treatment and informing familial recurrence. Information from a molecular diagnosis at both the variant-level and gene/pathway-level contributes to more personalized clinical care [7–11]. Increasingly, obtaining a molecular diagnosis can contribute to eligibility to participate in clinical trials that are underway to characterize the effects of targeted therapies for a number of different skeletal dysplasia conditions (https://clinicaltrials.gov). The genetically heterogeneous skeletal dysplasia conditions can show autosomal dominant, autosomal recessive, and X-linked modes of inheritance, and may also occur de novo [1]. Therefore, identifying a molecular diagnosis can inform genetic counseling about familial recurrence of the condition and aid family planning.

Historically, targeted variant or single gene testing for skeletal dysplasia conditions that were highest on the clinicians’ differential list were pursued sequentially until a molecular diagnosis was identified. This method of sequential testing can result in additional time and cost associated with a patient’s diagnostic odyssey and may deprioritize or overlook analysis of genes that have been newly associated with skeletal dysplasia conditions. Next-generation sequencing (NGS) technology has revolutionized both diagnostics and gene discovery within the last decade, resulting in > 90% (at least 425/461) of characterized skeletal dysplasia conditions being mapped to an associated genetic cause and molecular testing being implemented as a standard component of the diagnostic work-up [1, 5]. With the massively parallel capabilities of NGS technology, clinicians now commonly request simultaneous interrogation of multiple genes at once [12] using panel/targeted exome testing [13, 14] or whole exome sequencing [2, 15, 16] as part of a patient’s diagnostic work-up. Studies of postnatal patients with suspected skeletal dysplasia report diagnostic yields between ~ 20 and 39% for NGS-based multi-gene panel testing and whole exome analysis, with study populations ranging from 43 to 59 participants [13, 16]. Diagnostic yields reported in prenatal cases have been reported as high as ~ 55–89% [15], [2], [17], [14], with sample sizes ranging from 12 to 30 probands selected largely from single health systems.

To date, the molecular diagnostic utility of NGS multi-gene panel testing has not yet been characterized for a large, unselected population of individuals with suspected skeletal dysplasia. In this study, we retrospectively reviewed NGS multi-gene panel testing results from 543 individuals with suspected skeletal dysplasia to assess the diagnostic yield, performance in prenatal diagnostics, variant characteristics, and impact of copy number variation on molecular diagnostic utility.

## Results

A total of 543 patients were included in this study (Table 1). Patient samples were received via clinicians from multiple health systems across North America (46.0%, n = 250), Europe/Middle East/Africa (40.9%, n = 222), Asia Pacific (10.3%, n = 56), and Central and South America (2.8%, n = 15). The incidence of consanguinity was largely unknown for this population as it was not consistently reported across cases. Males and females (according to clinician report) were similarly represented in this cohort; however, patient sex was unreported for several fetal patients. The median patient age at time of testing was 4 years (range, fetal to 66 years). Most individuals (88.0%, n = 478) in this study received a comprehensive panel test that included up to 251 or 374 genes, while the remaining 12.0% (n = 65) received a core panel test of up to 113 genes.

**Table 1:** Patient demographics

	Number of individuals (n = 543)	Proportion of total cohort (%)
*Reported sex*		
Male	244	44.9
Female	228	42.0
Not reported	71	13.1
*Age at time of testing*		
Fetus	88	16.2
Infant (birth-age 2)	160	29.5
Childhood (ages 3–10)	132	24.3
Puberty (ages 11–18)	67	12.3
Young adult (ages 19–40)	68	12.5
Adult (ages 41–66)	28	5.2
*Panel received*		
Skeletal dysplasias core (up to 113 genes)	65	12.0
Comprehensive skeletal dysplasias and disorders (up to 251 genes)	269	49.5
Comprehensive growth disorders/skeletal dysplasias and disorders (up to 374 genes)	209	38.5

**Fig. 1:**
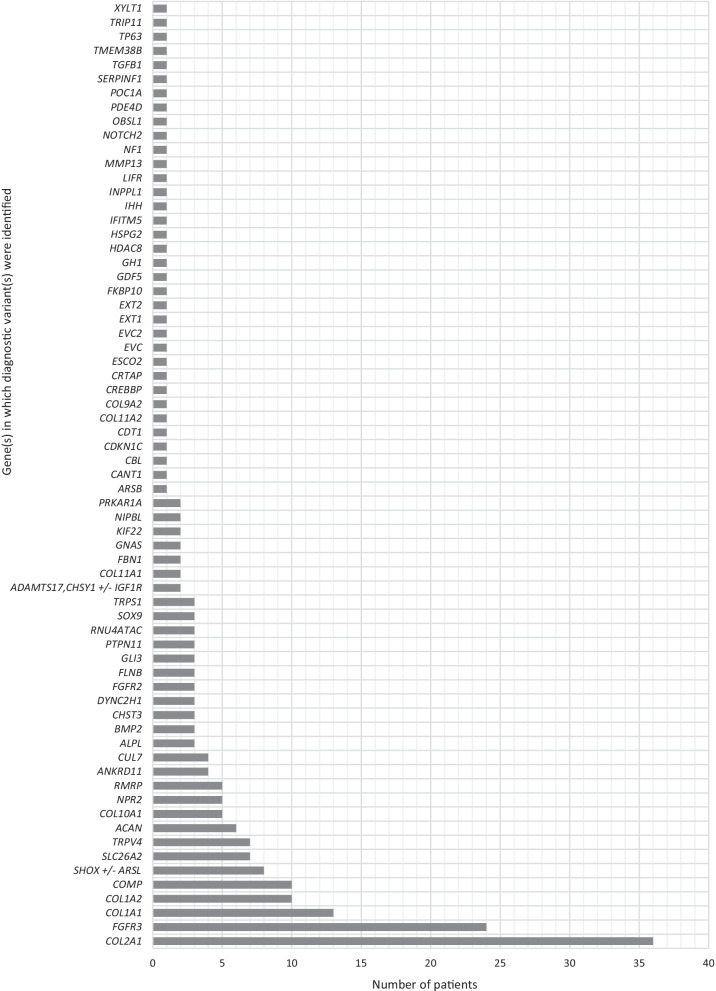
Numbers of patients with diagnostic variation by gene

A molecular diagnosis was established in 42.0% of patients (228/543). Diagnostic variants were identified in 71 genes (Fig. 1), with variation in nearly half of these genes (35/71, 49.3%) contributing to a molecular diagnosis for a single patient in this cohort. Overall, the most common genes in which molecular diagnoses were identified included: *COL2A1* (n = 36) associated with type II collagenopathies (MIM 120140); *FGFR3* (n = 24) associated with achondroplasia, thanatophoric dysplasia, hypochondroplasia, and other conditions such as FGFR-related craniosynostoses (MIM 134934); and *COL1A1* (n = 13) or *COL1A2* (n = 10) associated with osteogenesis imperfecta (OI; MIM 120150 and 120160, respectively). Together, variation in these four genes accounted for over one third of all molecular diagnoses across the cohort. Among patients with the *FGFR3*-related molecular diagnoses, four patients with clinical suspicion of achondroplasia (including one fetal case) received a confirmed molecular diagnosis of the *FGFR3* NP_000133.1:Gly380Arg variant. Many individuals with clinical suspicion of OI received molecular diagnoses in *COL1A1* and *COL2A1*. However, several patients showed molecular diagnoses in less common OI-related genes. For example, one child had two variants (NM_002615.5: c.271_279dupGCCCTCTCG p.(Ala91_Ser93dup), classified as pathogenic; and c.857_868delTGACCTTGATAG p.(Leu286_Glu290delins*), classified as likely pathogenic) in the *SERPINF1* gene, for which gene/pathway-guided treatment may be available. Of note, one heterozygous variant in the difficult-to-sequence exon 12 of the *ACAN* gene (NM_013227.3), c.5332C > T, p.(Gln1778*), was identified in a child with relative macrocephaly, short stature, hypoplastic chest, bowed forearms, and reduced elbow supination, pronation and extension. Additionally, 11.4% (36/315) of non-diagnostic patient reports had suspicious variants of unknown significance (VUS), including 10 cases in which one pathogenic or likely pathogenic variant and one rare, non-truncating VUS were reported in the same gene in association with an autosomal recessive condition (Additional file 1).

**Fig. 2:**
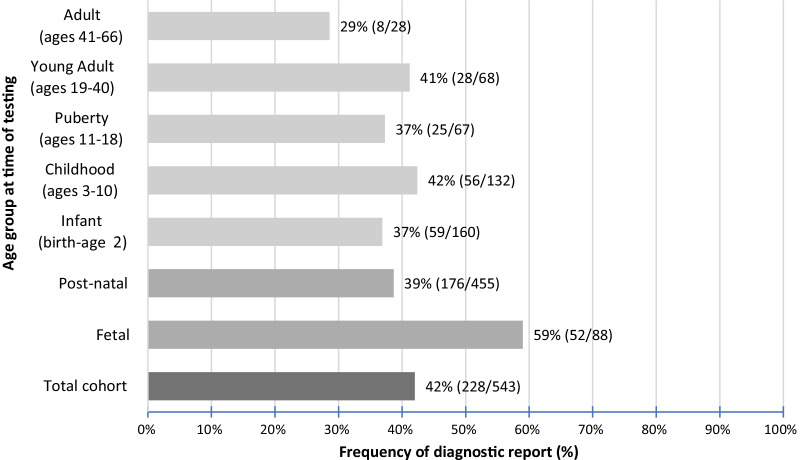
Diagnostic yield of next-generations sequencing-based multi-gene panel tests for suspected skeletal dysplasia by age at time of testing

Diagnostic yield was significantly higher among fetal samples (59.0%, n = 52/88) than postnatal samples (38.7%, n = 176/455; z = 3.55, *p* < 0.001) (Fig. 2). Diagnostic variants in fetal cases were identified across 18 genes (Additional file 2). In addition to disease-causing variation in the *FGFR3* (n = 8), *COL2A1* (n = 7), *COL1A1* (n = 8), and *COL1A2* (n = 6) genes, fetal molecular diagnoses were also more common in the *DYNC2H1* (n = 3) and *SOX9* (n = 3) genes associated with autosomal recessive short-rib thoracic dysplasia (MIM 613091) and campomelic dysplasia (MIM 114290), respectively. Fetal molecular diagnoses were also identified due to variation in the *ALPL, COL11A1, COL11A2, CRTAP, ESCO2, EVC2, FGFR2, FLNB, GLI3, RMRP, SLC26A2,* and *TRIP11* genes.

Thirteen diagnostic CNVs were reported (Table 2), representing 5.7% of total diagnostic findings. Diagnostic CNVs included intragenic (n = 3), single gene (n = 3), and multigenic (n = 5) variation in addition to whole-chromosome aneuploidy (n = 2). The three diagnostic intragenic CNVs were all copy number losses that encompassed just one or two coding exons in the gene. The smallest diagnostic CNV identified in this cohort was a 241-base pair (bp) homozygous single-exon deletion in the *TMEM38B* gene (NM_018112.3: exon 4) in an infant with reported hypotonia, developmental delay, blue sclera, consanguinity, and a family history of clinically diagnosed osteogenesis imperfecta (Study ID 324, Table 2. Truncating biallelic variants in the *TMEM38B* gene are an established mechanism causing autosomal recessive osteogenesis imperfecta type XIV (MIM 615066). In addition to a heterozygous, pathogenic single nucleotide variant (NM_014780.4: c.4717C > T, p.Arg1573*), a 458-bp single-exon deletion was identified in the *CUL7* gene associated with autosomal recessive 3-M syndrome (MIM 273750) in a child with macrocephaly, overall short stature, thin tubular bones, and short rib/small thorax entity (Study ID 247, Table 2).

**Table 2:** Diagnostic copy number variants identified among patients undergoing next-generation sequencing-based multi-gene panel testing for suspected skeletal dysplasia

Study ID	Phenotype	Chrom	Event	Copy number	OMIM morbid gene(s)	Size category	Minimum size (bp)	Classification
324	Hypotonia, developmental delay, blue sclera. Family history of OI. Consanguinity reported	9	Loss	0	***TMEM38B***	Intragenic	241	Pathogenic
247	Macrocephaly, short stature, thin tubular bones, small thorax	6	Loss	1	***CUL7***	Intragenic	458	Likely pathogenic
096	Skeletal dysplasia consistent with Stickler syndrome or brachyolmia	1	Loss	1	***COL11A1***	Intragenic	827	Likely pathogenic
435	Short stature, dysmorphic facies	X	Loss	1	***SHOX***	Single gene	20,429	Pathogenic
460	Short stature, markedly reduced limb length, scoliosis, chronic musculoskeletal pain	X	Loss	0	***SHOX***	Single gene	20,429	Pathogenic
226	Short stature, markedly reduced limb length. Father with short limbs	X	Loss	1	***SHOX***	Single gene	324,729	Pathogenic
516	Short stature, dysmorphic facies, speech delay	20	Loss	1	***BMP2***, *MCM8, FERMT1*	Multigenic	827,196	Pathogenic
454	Disproportionated short stature, hypermobility, delayed bone age, abnormal epiphyses	15	Loss	1	***ADAMTS17, CHSY1***, IGF1R *MEF2A, CERS3, LINS1, ALDH1A3*	Multigenic	3,343,316	Pathogenic
456	Disproportionate short stature	15	Loss	1	***ADAMTS17, CHSY1, IGF1R,*** *MEF2A, CERS3, LINS1, ALDH1A3*	Multigenic	3,764,795	Pathogenic
257	Rhizomelic short stature, relative macrocephaly	X	Loss	1	***SHOX***, ***ARSL***, *CSF2RA, XG, NLGN4,*	Multigenic	5,774,581	Pathogenic
540	Rhizomelic short stature, relative microcephaly, difficulty pronating and supinating arms. Family history if similar features in two first-degree relatives and others	X	Loss	1	***SHOX, ARSL***, *CSF2RA, XG, NLGN4, STS*	Multigenic	6,683,360	Pathogenic
023	Multiple congenital anomalies including skeletal abnormality of the radius and abnormal hands	18	Gain	3	Whole chromosome	Aneuploidy	N/A	Pathogenic
233	Short stature, possible reduced femur length, coarse facial features	X	Loss	1	Whole chromosome	Aneuploidy	N/A	Pathogenic

Bolded font represents the genes included on the multi-gene panel received by this patient that are encompassed within the CNV (see Additional file 3)

Five CNVs involved loss of the *SHOX* gene in the pseudoautosomal region of the X and Y chromosomes (Table 2). Functional loss or haploinsufficiency of *SHOX* causes various short stature conditions and disturbed bone development with wide phenotypic spectrum within and among affected families, including pseudoautosomal dominant disorders Leri-Weill dyschondrosteosis (MIM 127300) and idiopathic familial short stature (MIM 300582) on the mild end and recessive Langer mesomelic dysplasia on the more severe (MIM 249700). One young adult male patient (Study ID 460, Table 2) showed no copies of the *SHOX* gene and a phenotype including short stature, reduced arm and leg length (proportions not specified), scoliosis, limited range of motion of arms, knees, and spine, and chronic musculoskeletal pain that interfered with basic daily activities. Prior to this genetic analysis, the patient and several of his living family members had been clinically diagnosed with hypochondroplasia despite no disease-causing variation in the *FGFR3* gene being identified through previous genetic testing at a different laboratory. Additionally, two unrelated female patients in this study with rhizomelic short stature and relative macrocephaly show multigenic *SHOX-ARSL* gene deletions.

## Discussion

To our knowledge, this represents one of the largest studies of molecular diagnostics within an unselected population of patients with suspected skeletal dysplasia. The overall diagnostic yield of 42% demonstrates the utility of NGS-based panel testing in identifying molecular diagnoses for individuals with skeletal dysplasia in this cohort. Molecular diagnostic yields can vary substantially based on case selection or study recruitment (including known consanguinity and average age of participants at time of study/clinical ascertainment), effect size of the examined cohort, technical aspects of the genetic testing platforms (such as the inclusion of copy number variant analysis), analysis structure of genetic data (targeted multi-gene vs unbiased exome/genome approach, proband-only vs inclusion of family members for segregation/inheritance analysis), and prior clinical and genetic work-up, among many other factors. Yields tend to be higher in studies with fewer participants, consanguineous populations, cohorts with a younger average age of participants at time of clinical ascertainment, and populations more naïve to prior diagnostic work-up [18]. Both the fetal (58.0%) and postnatal (38.9%) diagnostic yields in this study are consistent with diagnostic yields reported in respective studies of molecular diagnostics among patients with skeletal dysplasia using NGS methods including multi-gene panel testing and exome/genome analysis [15], [2], [17], [13], [16], [14],however, it is important to note that this study characterizes an unselected population with a wide postnatal age distribution and larger number of participants than other published studies. Additionally, the diagnostic yields in these studies such as ours may increase upon continued study of suspicious VUSes. Some of these suspicious VUSs would be reclassified to likely pathogenic if family segregation data showed de novo status for heterozygous variants in genes associated with autosomal dominant conditions, or if variants are confirmed in trans in genes associated with autosomal recessive conditions. Family studies in these cases were not available at the time of analysis but may be pursued later to contribute to evidence supporting variant classification.

Molecular diagnoses in fetal samples spanned 18 genes, showing significant genetic heterogeneity among affected individuals presenting prenatally. Han et al. [2] reports that variants in *FGFR3, COL1A1, COL1A2* accounted for 66.7% (18/27) of total diagnosed fetal cases, while this study’s findings show 40.0% (22/55) of fetal molecular diagnoses resulted from variants in these three genes. Therefore, most fetal molecular diagnoses in this study involved variants in genes less commonly identified in prenatal testing, emphasizing the value of testing multiple genes simultaneously when phenotypic overlap between differential diagnoses is significant. Given the time pressure often associated with fetal molecular diagnostics in addition to challenges with accurate phenotyping depending on access to diagnostic imaging and expertise, it may be most prudent to perform a single comprehensive panel test rather than potentially serial single gene tests, if differential clinical diagnoses show significant genetic heterogeneity [15].

Copy number variants, including exon-level intragenic deletions, were significant contributors to diagnostic yield, representing 5.7% of total diagnostic findings. Assays that allow for simultaneous analysis of both single nucleotide variation and CNVs can contribute to molecular diagnoses in which biallelic variation is the mechanism of disease, as identified in one patient in this cohort with *CUL7*-related autosomal recessive 3-M syndrome.

The inclusion of difficult-to-sequence genomic regions in comprehensive molecular testing for individuals with suspected skeletal dysplasia and growth disorders is essential to maximize diagnostic benefit. Variation in both the *ACAN* gene and the *SHOX* gene contributed to the molecular diagnostic yield in this patient cohort. Because exon 12 of the *ACAN* gene encompasses a variable number tandem repeat (VNTR), single nucleotide variation in this region is difficult to call via NGS methodology and therefore can be excluded from analysis [19]. A diagnostic single nucleotide variation in exon 12 was identified for one patient in this cohort, showing the importance of targeted coverage of this region with an intentional orthogonal testing strategy for variants meeting criteria for confirmation. Additionally, phenotypic variability is appreciated in patients with *SHOX* deficiency and contiguous gene deletions involving *SHOX* are common and result in more complex phenotypes. With NGS-based methods, this region is difficult to align and call smaller variants, owing to segmental duplication with > 98% homology seen between *SHOX* exons 2–6 (NM_000451.3) and other areas of the genome. Despite this homology, bioinformatic copy number analyses are possible with robust CNV calling algorithms, given the frequency of *SHOX*-related molecular diagnoses identified in this study, these analyses should not be excluded from molecular diagnostics of individuals with suspected skeletal dysplasia and growth disorders.

Molecular analyses are an important part of a patient’s diagnostic journey and care plan, alongside clinical and radiographic assessments. Both variant-level and gene/pathway-level information from molecular diagnoses identified in this cohort may lead to more personalized treatment. For example, variant-level information is useful when considering the use of C-type natriuretic peptide (CNP) analogue to aid in growth of long bones, as individuals with achondroplasia and a confirmed molecular diagnosis of the *FGFR3* NP_000133 0.1:Gly380Arg substitution have shown increased annualized growth velocity in clinical trials data [10]. Also, gene/pathway-level information helps to guide the use of bisphosphonates to inhibit osteoclast activity in individuals with OI. Bisphosphonate therapy has been shown to increase bone mineral density in the majority of individuals with autosomal dominant OI who show causative variation in the *COL1A1* or *COL1A2* genes [7], however, individuals with causative variants in other genes/pathways often do not respond to this therapy. For example, an individual with a molecular diagnosis involving variation in the *SERPINF1* gene associated with autosomal recessive OI type 6 who shows a poor response to bisphosphonate therapy [11], but may respond to denosumab, a human monoclonal antibody against Rankl [9].

The high molecular diagnostic yield in pre- and post-natal populations highlights a potential benefit of pursuing comprehensive molecular testing early in the diagnostic process for individuals with suspected skeletal dysplasia: this approach may both shorten the diagnostic odyssey and benefit therapy. Once a molecular diagnosis has been obtained, the patient can be referred to appropriate specialists for additional clinical correlation and, if appropriate, management or therapy guidance. This may contribute to a more efficient diagnostic process, as opposed to delaying molecular testing for an individual with suspected skeletal dysplasia until after a full clinical specialty work-up has been completed. In this large cohort, almost half of the molecular diagnoses were in unique genes. This finding highlights the value of NGS-based panel testing in interrogating multiple genes simultaneously, given the known genetic heterogeneity and substantial phenotypic overlap between skeletal dysplasia conditions.

Limitations of these data include the evolving definition of the assays used and gene content included over time. Specificity and sensitivity of variant detection changed with the different sequencing platforms and assays that were introduced during the period of study. The number of genes included on the panels also changed due to evolving information about relationships between genes and disease. Because the sensitivity and specificity of the assays used were optimized and the number of genes interrogated generally increased over time, patients with non-diagnostic reports who received testing earlier in the study may benefit from updated testing. The average turnaround time for fetal samples was not analyzed in this study, although this is recognized as an important factor in diagnostic utility for fetal samples [15]. This study could be expanded to include individuals pursuing diagnostic genetic testing for indications of non-syndromic short stature or proportional growth disorders, given the possible phenotypic overlap among these individuals and those with skeletal dysplasia [8]. Characterizing the frequency of molecular diagnoses by patient phenotypic features or age at first clinical presentation is not possible, owing to the limited phenotype and age at onset information available for analysis. This work is encouraged among others with similar interests.

## Conclusions

This study demonstrates the molecular diagnostic utility of panel testing for individuals with a suspected skeletal dysplasia or growth disorder, with a particularly high diagnostic yield seen in prenatal cases. Pursuing comprehensive panel testing with high-resolution CNV analysis can provide a diagnostic benefit, given the considerable phenotype overlap amongst skeletal dysplasia conditions.

## Methods

Clinical reports of consecutive patients who underwent diagnostic genetic testing at Blueprint Genetics (a CLIA-certified diagnostic laboratory) were examined. All patient samples were submitted for diagnostic testing with an indication of a suspected skeletal dysplasia or growth disorder. Clinicians summarized and reported patient phenotypic information via the laboratory’s test requisition form. Informed parental or personal consent for diagnostic genetic testing was obtained for all patients included in this study. This subsequent retrospective study of patient data was given the status of exempt by WCG Institutional Review Board.

Genetic testing was performed using NGS methods at the Blueprint Genetics laboratory. For 56 patients, NGS was performed using the OS-Seq™ (oligonucleotide-selective sequencing) NGS method [20] on the NextSeq™ sequencing system (Illumina). In this analysis, the median sequencing depth was 177X and an average of 99.64% of target nucleotides were covered with > 20 × sequencing depth. For 378 patients, NGS was performed using an in-house tailored IDT-based clinical exome sequencing platform (Integrated DNA Technologies) performed on the NovaSeq™ sequencing system (Illumina). In this analysis, the median sequencing depth was 196X and 99.93% of target nucleotides were covered with > 20 × sequencing depth. For 109 patients, NGS was performed using an in-house tailored Twist-based clinical exome sequencing platform (Twist Biosciences) performed on the NovaSeq™ sequencing system (Illumina). In this analysis, the median sequencing depth was 215X and 99.74% of target nucleotides were covered with > 20 × sequencing depth. This platform included custom oligonucleotides targeting known disease-associated non-coding/deep intronic variants (Additional file 3).

The patients included in the study received one of three NGS-based panel tests: the ~ 113-gene Skeletal Dysplasia Core Panel; the ~ 251-gene Comprehensive Skeletal Dysplasias and Disorders Panel; or the ~ 374-gene Comprehensive Growth Disorders/Skeletal Dysplasias and Disorders Panel (Additional file 3). These panels are considered nested, as all the genes in the smaller panels are included in the larger panels. Gene content changed over time: the Skeletal Dysplasias Core Panel contained between 108 and 113 genes; the Comprehensive Skeletal Dysplasias and Disorders Panel contained between 187 and 252 genes; and the Comprehensive Growth Disorders/Skeletal Dysplasias and Disorders Panel contained between 332 and 374 genes. The lists of genes provided in Additional file 3 represent the most genes possibly interrogated per panel for any given patient.

Panel testing included both sequence and copy number variant (CNV) analyses of NGS data, targeting coding exons and ± 20 bp from the intron/exon boundaries. Single nucleotide and indel variant calling were performed using tailored GATK algorithms (Sentieon) for nuclear DNA. Copy number analysis was performed bioinformatically from NGS data using two variant-calling algorithms, including both CNVkit [21] and a proprietary method specific for small, exon-level CNVs. Sequence reads of each sample were mapped to the human reference genome (GRCh37/hg19). The median sequencing depth and coverage across the target regions for the tested sample were calculated based on MQ0 aligned reads. Bi-directional Sanger sequencing or digital polymerase chain reaction (ddPCR) methods were used to orthogonally confirm variants that did not meet our rigorous internal sequencing quality score thresholds and other laboratory criteria. Copy number variants were confirmed if they are less than 10 exons (heterozygous) or less than 3 exons (homozygous/hemizygous) in size or have not been confirmed at least three times previously at our laboratory. Variants in areas with high homology and with known pseudogenes were flagged for further attention. All variants of interest were visually inspected for quality by a geneticist in Integrated Genomics Viewer (IGV), and variants with questionable quality upon inspection were sent for confirmation at the discretion of the reporting geneticists and/or lab director. Additionally, all variants reported on reports from prenatal samples were orthogonally confirmed. The sequence variant analysis and CNV analysis pipelines were validated in the CLIA-certified, CAP-and ISO15189-accredited Blueprint Genetics diagnostic laboratory. Variant classification and reporting were performed in accordance with Association for Molecular Pathology/American College of Molecular Genetics and Genomics guidelines [22].

For the purposes of this study, a molecular diagnosis was defined as the identification of pathogenic (P) or likely pathogenic (LP) variant(s) consistent with the patient’s reported phenotype and with known associated disease inheritance. Suspicious variants of unknown significance (VUS) were defined as having all the following characteristics: (1) a strong and specific correlation between the gene and patient’s phenotype, (2) the variant being novel or extremely rare in the Genome Aggregation Database (gnomAD) control cohorts, and (3) in silico predictions supporting pathogenicity or the amino acid position in question being highly conserved in mammals and evolutionary more distant species, suggesting that the position does not tolerate variation. Cohort demographics and diagnostic yields were calculated using descriptive statistics and z-tests were used to assess for significance, assuming binomial distribution.

## Supplementary Information

**Additional file 1**. Variants in non-diagnostic cases with suspicious VUSes.

**Additional file 2**. Diagnostic prenatal variants.

**Additional file 3**. Panel gene content and targeted non-coding variants.

## Data Availability

The datasets generated and analyzed during the current study are available from Blueprint Genetics upon reasonable request. Restrictions apply to the availability of these data as they contain sensitive information that could compromise patient privacy/consent and so are not publicly available.

## Abbreviations

AD: Autosomal Dominant
AR: Autosomal Recessive
CNV: Copy Number Variant/Variation
HET: Heterozygote
HOM: Homozygote
LP: Likely Pathogenic
MOI: Mode of Inheritance
NGS: Next-generation sequencing
P: Pathogenic
VUS: Variant of Unknown Significance
