# Correction to: Diagnostic utility of next-generation sequencing-based panel testing in 543 patients with suspected skeletal dysplasia

**DOI:** 10.1186/s13023-022-02242-8

**Published:** 2022-02-17

**Authors:** Alicia Scocchia, Tiia Kangas-Kontio, Melita Irving, Matti Hero, Inka Saarinen, Liisa Pelttari, Kimberly Gall, Satu Valo, Johanna M. Huusko, Jonna Tallila, Johanna Sistonen, Juha Koskenvuo, Tero-Pekka Alastalo

**Affiliations:** 1 Blueprint Genetics Inc, Seattle, WA USA; 2 Blueprint Genetics Oy, Espoo, Finland; 3 Department of Clinical Genetics, Guy’s and St. Thomas’ NHS Trust, London, UK; 4 New Children’s Hospital, Pediatric Research Center, Helsinki University Hospital, Helsinki, Finland

## Correction to: Orphanet J Rare Dis (2021) 16:412 10.1186/s13023-021-02025-7

Following the publication of the original article [1] the authors noticed an inconsistency between the following sentences:

“Diagnostic yield was significantly higher among fetal samples (58.0%, n = 51/88) than postnatal samples (38.9%, n = 177/455; z = 3.32, *p* < 0.0009)” in the “Results” section of the “Abstract”

and

“Diagnostic yield was significantly higher among fetal samples (58.0%, n = 51/88) compared to postnatal samples (38.9%, n = 177/455; z = 3.32, *p* < 0.0001)” in the “Results” section of the main text.

The correct information is the one reported in Figure 2: “Diagnostic yield was significantly higher among fetal samples (59.0%, n = 52/88) than postnatal samples (38.7%, n = 176/455; z = 3.55, *p* < 0.001)”.

The original article has now been updated with the “Results” section of the “Abstract” and “Results” section of the main text having been corrected as per Figure 2.
